# Distribution of *Blastocystis* subtypes isolated from humans from an urban community in Rio de Janeiro, Brazil

**DOI:** 10.1186/s13071-017-2458-0

**Published:** 2017-10-25

**Authors:** Carolina Valença Barbosa, Rosemary de Jesus Batista, Ricardo Pereira Igreja, Claudia Masini d’Avila Levy, Heloisa Werneck de Macedo, Helena Lúcia Carneiro Santos

**Affiliations:** 1 Laboratório de Estudos Integrados em Protozoologia, Instituto Oswaldo Cruz/FIOCRUZ, Av. Brasil, 4365 Pavilhão Arthur Neiva, Rio de Janeiro, 21.045-900 Brazil; 2 Departamento de Ginecologia e Obstetrícia, IFF/Fiocruz, Rio de Janeiro, Brasil, Av. Rui Barbosa, 716, Flamengo, Rio de Janeiro, 22250-020 Brazil; 3 Departamento de Medicina Preventiva da Faculdade de Medicina da UFRJ , Av. Carlos Chagas Filho, 373, Edifício do Centro de Ciências da Saúde Bloco K, 2° andar, Sala 49 - Cidade Universitária - Ilha do Fundão, Rio de Janeiro, 21.941-902 Brazil; 4 Laboratório de Parasitologia do Departamento de Patologia, Hospital Universitário Antônio Pedro/Universidade Federal Fluminense, Niterói, RJ, Rua Marquês do Paraná, 303. 4º andar, sala 12, Niterói, Rio de Janeiro, 24033 900 Brazil

**Keywords:** *Blastocystis*, PCR, Subtypes, Epidemiology

## Abstract

**Background:**

*Blastocystis* is a cosmopolitan protist parasite found in the human gastrointestinal tract and is highly prevalent in developing countries. Recent molecular studies have revealed extensive genetic diversity, which has been classified into different subtypes (STs) based on sequence analysis of small subunit ribosomal RNA gene. *Blastocystis* is one of the most common fecal parasites in Brazil, but the diversity of subtypes remains unknown in the country. This study aimed to determine the distribution of *Blastocystis* STs in an urban community in Duque de Caxias, Rio de Janeiro, Brazil.

**Methods:**

A total of 64 stool samples positive for *Blastocystis* in Pavlova’s medium were subtyped by PCR and sequenced using primers targeting the small subunit rRNA gene, in addition to phylogenetic analysis and subtype-specific PCR using sequence-tagged-site (STS) primers.

**Results:**

*Endolimax nana* (14%), *Entamoeba* complex (10.5%), *Taenia* sp. (0.6%), *Trichuris trichiura* (1.3%) and *Enterobius vermicularis* (1.3%) were detected in *Blastocystis-*positive samples. Of the 64 samples tested by PCR/DNA sequencing, 55 were identified as ST1 (42%), ST3 (49%), ST2 (7%) and ST4 (2%), and the presence of mixed ST (ST1 + ST3) infection was detected in nine samples (14%).

**Conclusions:**

DNA sequencing and phylogenetic analysis of Brazilian *Blastocystis* isolates identified four different subtypes. To our knowledge, this study provided the first genetic characterization of *Blastocystis* subtypes in an urban area of Rio de Janeiro, Brazil. We also identified ST4 for the first time in Brazil. Further studies are necessary to determine the distribution of STs across human populations in Rio de Janeiro.

## Background

*Blastocystis* spp. are strictly anaerobic unicellular organisms that inhabit the gastrointestinal tract of humans as well as other mammals, birds, amphibians, reptiles, fish, arthropods and annelids [1–9]. *Blastocystis* is a cosmopolitan enteric protist estimated to infect over one billion people worldwide [10]. However, its prevalence rates vary widely between countries and between regions within the same country [11, 12], possibly reaching 30% in industrialized countries and 30–76% in developing nations [13–18]. In the latter, high prevalence rates may be directly related to educational deficits, poor socioeconomic status, and sanitary conditions [19–22]. Nevertheless, high rates of infection are also observed in developed countries, especially among individuals who work with animals [6, 7, 23]. Previous studies have reported that the transmission of *Blastocystis* is fecal-oral, and as a result the protist was included in the Water Sanitation and Health program of the World Health Organization [24].

Although *Blastocystis* is an emerging intestinal parasite [25], its pathogenic potential remains unclear [24]. Diagnostic limitations, extensive genetic diversity, poor understanding of the host-parasite relationship, and the lack of an experimental model represent major bottlenecks in elucidating the clinical significance and pathogenesis of *Blastocystis*. After infection (blastocystosis), not all persons develop the clinical disease, for which common symptoms include chronic or acute diarrhea, abdominal pain, flatulence, anorexia, nausea and vomiting [26–28]. One case of enteroinvasive blastocystosis diagnosed by histopathological analysis of biopsies showed *Blastocystis* in ulcers in the cecum, transverse colon and rectum of an immunocompetent individual, indicating that the parasite has invasive potential [29]. In addition, a case of appendicitis associated with appendix lumen obstruction caused by *Blastocystis* has also been reported [30]. *Blastocystis* subtype 3 (ST3) was detected in the appendix, recto-uterine pouch, and peritoneal fluid of a nineyear-old child [31] and in splenic cysts of an immunocompetent woman [32]. Other studies have reported extraintestinal manifestations including arthritis (with full resolution of clinical symptoms after metronidazole treatment) and recurrent episodes of arthritis in immunocompetent individuals [33]. Additionally, several studies suggest an association of *Blastocystis* with urticaria and skin lesions in immunocompetent individuals [34, 35]. These findings provide strong evidence of the ability of *Blastocystis* to migrate to extraintestinal sites and of its pathogenic potential. Moreover, some epidemiological surveys have suggested a strong association between irritable bowel syndrome (IBS) and *Blastocystis*, but this association has been questioned [36, 37].

Molecular analyses of the small-subunit ribosomal RNA have revealed extensive genetic diversity in the species. To date, 17 subtypes have been described (ST1-ST17), nine of which (ST1-ST9) are found both in humans and animals, while the others are exclusively found in animals [8, 38]. These subtypes have not been characterized as species, have low host specificity, and were isolated from humans and various animal hosts [39]. In humans, the most frequently detected subtype is ST3, followed by ST1, ST2, and ST4, whereas ST5-ST9 are rarely found [9, 40]. Genetic diversity varies dramatically among *Blastocystis* subtypes, which are not only separated by significant genetic distance [7, 39] but also differ in levels of intra-subtype variation [41]. Thathaisong et al. [42] detected polymorphisms in *Blastocystis* ST1 and created ST1/variant, while Melloni et al. [43] observed substantial intra-ST diversity in ST3 isolates and genetic homogeneity in ST4 isolates.

So far, studies have revealed the existence of genetic variability [44–47]. However, studies on *Blastocystis* diversity remain scarce in Brazil, where only three studies have been conducted to date [14, 48, 49]. To address this shortcoming, systematic studies focused on characterizing different isolates are necessary. In this study we report the molecular identification of four subtypes of *Blastocystis* in a Brazilian population. This study is the first to thoroughly investigate the diversity of *Blastocystis* subtypes in an urban community in Rio de Janeiro, Brazil. Our findings may be relevant to identifying sources of infection and potential routes of transmission of the parasite.

## Methods

A coproparasitological study was conducted at the Center for Psychosocial Care (CAPS), a day-care hospital for the mentally ill in Duque de Caxias, Rio de Janeiro, Brazil.

Participation was voluntary and people could withdraw from the study at any time. To guarantee anonymity, each study participant was given a unique identification number. At the end of the study, all participants identified with enteric parasitic pathogens received proper antiparasitic drugs for treatment. Treatment was not offered to individuals infected with *Blastocystis*, because its pathogenic potential is still a topic of debate.

After written consent was obtained, fecal material from each participant was collected in a flask and transported to the laboratory. Unpreserved stool samples were requested and collected. A total of 180 unpreserved stool samples were collected at home from non-selected patients (*n* = 80) and their family members (*n* = 100), and kept at 4 °C and transported in ice packs to the Parasitology Laboratory at Oswaldo Cruz Foundation. Fresh unpreserved stool samples were fractionated into two aliquots upon receipt.

For parasitological analysis, one aliquot was processed using a spontaneous sedimentation technique (the Lutz technique or the Hoffman, Pons and Janer technique). Stool samples diluted in water were filtered through a gauze strip into a conical cup and subsequently submitted to sedimentation in tap water for 2 h [50]. The concentrated sample was analyzed by wet mounts and stained through an iodine staining procedure. In this study, we did not search for viruses or bacteria.

Direct cultivation of *Blastocystis* from the aliquot of stool samples was performed using Pavlova’s medium (1.29 g/l of sodium phosphate dibasic, 0.42 g/l of potassium phosphate monobasic, 7.27 g/l of sodium chloride and 1.46 g/l of yeast extract) supplemented with 10% adult bovine serum (ABS) and penicillin-streptomycin (1000 IU/ml and 500 μg/ml, respectively) and incubated at 37 °C [51]. Screening of *Blastocystis* cultures was performed using a standard light microscopy in the first seven days of incubation. When typical forms of the parasite were observed, *Blastocystis* culture aliquots were frozen at -20 °C until used for DNA extraction.

### Molecular characterization

Total DNA of each isolate was extracted from each culture of *Blastocystis* suspension using the Qiamp DNA Stool Mini Kit (Qiagen, Valencia, CA, USA) according to manufacturer’s recommendations. DNA was stored at -20 °C until use. To amplify segments from an approximately 500 bp fragment of genus-specific small subunit ribosomal DNA (SSU rDNA), we used the following primers: forward Blast 505-532 (5′-GGA GGT AGT GAC AAT AAA TC-3′) [52] and reverse Blast 998-1017 (5′-TGC TTT CGC ACT TGT TCA TC-3′), following the protocol described by Santin et al. [8]. The PCR reaction was performed in a final volume of 50 μl and each reaction contained 100 mM of Tris-HCl (pH 9.0), 500 mM of KCl, 1.5 mM of MgCl_2_, 200 μM of each dATP, dGTP, dCTP, and dTTP; 0.2 μM of primer; 1.5 U of *Taq* DNA polymerase (Invitrogen Life Technologies, Carlsbad, CA, USA), 0.05% of bovine serum albumin (BSA); and 5 μl of the DNA sample. Cycle conditions were an initial activation step at 95 °C for 5 min, followed by 35 cycles at 95 °C for 30 s, 55 °C for 30 s, and 72 °C for 2 min, with a final extension step at 72 °C for 7 min. PCR amplification reactions were performed in a Veriti™ 96-well thermal cycler (AB Applied Biosystems, Foster City, CA, USA). PCR products were electrophoresed in 1.5% agarose gels in a tris-borate EDTA buffer, stained with Gelred (Biotium Inc., Hayward, CA, USA), and photographed under UV transillumination. Amplicons were purified using the Wizard® SV gel and PCR Clean-Up System kit (Promega, Madison, WI, USA) and sequenced in both directions using the PCR primers. DNA cycle sequencing reactions were performed using the BigDye® Terminator v.3.1 Cycle Sequencing Kit (Applied Biosystems) and loaded in the ABI 3730 Sequencing Platform. Bi-directional sequences were assembled and edited using SeqMan (DNASTAR software package, DNASTAR Inc., Madison, WI, USA). DNA sequences of *Blastocystis* subtypes were downloaded from GenBank and a multiple sequence alignment was performed using the ClustalW algorithm of MEGA software version 6.0 [53]. Bayesian Inference (BI), Maximum Likelihood (ML), and Neighbor-joining analyses of SSU rDNA data were conducted to explore the relationships between taxa using MrBayes [54], PHYML [55] and MEGA software [53], respectively. Nucleotide substitution models were chosen based on the Akaike Information Criterion in jModelTest [56] and MrModeltest [57]. ML analysis was conducted using the Hasegawa-Kishino-Yanov Model with gamma distribution (HKY + G) [58] with four parameters and unequal base frequencies including rate variation among sites (-lnL = 24,599.056), which was predicted as the best model of nucleotide substitutions by the Akaike Information Criterion (AIC). For Bayesian analysis, the HKY + G model was determined by the Bayesian Information Criterion (BIC) as the best-fit substitution model. *Proteromonas lacerate* (U37108) was used as an out-group [59, 60].

Subtype sequences with mixed traces indicative of mixed infections were analyzed by PCR using subtype-specific sequence-tagged-site (STS) primers (Table 1), according to Yoshikawa et al. [61].

**Table 1:** Sequence-tagged site (STS) primer sets used in this study targeting SSU rRNA gene of *Blastocystis*

Primer pair	Target subtype	Primer type	Sequence (5′-3′)
SB83	ST1	F	GAAGGACTCTCTGACGATGA
		R	GTCCAAATGAAAGGCAGC
SB340	ST2	F	TGTTCTTGTGTCTTCTCAGCTC
		R	TTCTTTCACACTCCCGTCAT
SB227	ST3	F	TAGGATTTGGTGTTTGGAGA
		R	TTAGAAGTGAAGGAGATGGAAG
SB337	ST4	F	GTCTTTCCCTGTCTATTCTGCA
		R	AATTCGGTCTGCTTCTTCTG
SB336	ST5	F	GTGGGTAGAGGAAGGAAAACA
		R	AGAACAAGTCGATGAAGTGAGAT
SB332	ST6	F	GCATCCAGACTACTATCAACATT
		R	CCATTTTCAGACAACCACTTA
SB155	ST7	F	ATCAGCCTACAATCTCCTC
		R	ATCGCCACTTCTCCAAT

## Results

A total of 180 participants aged 1–85 years (80 patients and 100 patients’ family members) provided adequate stool samples for the study; 105 (58.3%) were male (mean age: 52 years) and 75 (41.7%) were female (mean age: 48 years). Overall, based on light microscopy examination, 82 (45%) participants were found to be infected with one or more species of intestinal parasites. Of the 180 samples tested by the culture method for the presence of *Blastocystis*, 64 (35.5%) were positive, whereas microscopy examination was less effective in detecting *Blastocystis*, with only 49 (27%) samples showing positive results. The frequency of *Blastocystis* infections was 32.5% (26/80) among patients and 38% (38/100) in family members. Among the 64 *Blastocystis*-positive participants, 18.3% (33/180) of individuals (14 patients and 19 family members) presented poly-parasitism. *Endolimax nana* (14%), *Entamoeba* complex (10.5%), *Taenia* sp. (0.6%), *Trichuris trichiura* (1.3%) and *Enterobius vermicularis* (1.3%) were detected in the *Blastocystis-*positive samples. Of the 31 participants singly infected with *Blastocystis*, only five family members reported symptoms: two individuals reported intermittent episodes of diarrhea, while the other three respectively presented flatulence, bloating and abdominal discomfort.

### Typing of *Blastocystis*

APCR amplicon of approximately 500 bp of the SSU rDNA was successfully generated in all 64 samples. DNA sequencing revealed single-subtype infection in 55 isolates. Those 55 sequences showed a high identity (97–100%) to the reference sequences previously published by GenBank, and were identified as four distinct subtypes: ST1, ST2, ST3 and ST4. *Blastocystis* ST3 was the most prevalent subtype, followed by ST1, ST2 and ST4 (Table 2). In the remaining nine samples, sequence chromatogram analysis revealed the presence of double traces and mixed-subtype infection, and accordingly a PCR with subtype-specific sequence-tagged-site (STS) primers revealed the presence of both ST1 and ST3 (Table 2). In relation to the distribution of reported symptoms, ST2 was found in two family members with intermittent diarrheal episodes, whereas ST1, ST2 and ST3 were identified in three relatives of patients who respectively reported flatulence, bloating and abdominal discomfort.

**Table 2:** *Blastocystis* subtypes identified in the present study

Subtype	No. of isolates	Isolate code and GenBank accession number
ST1	23	RTDC1 (KU892242); RTDC4 (KU892241); RTDC5 (KU892222); RTDC8 (KU892243); RTDC17 (KU892244); RTDC27 (KU892225); RTDC29 (KU892233); RTDC32 (KU892227); RTDC35 (KU892237); RTDC36 (KU892229); RTDC37 (KU892235); RTDC38 (KU892236); RTDC39 (KU892230); RTDC40 (KU892238); RTDC41 (KU892231); RTDC42 (KU892240); RTDC49 (KU892232); RTDC50 (KU892226); RTDC52 (KU892239); RTDC55 (KU892234); RTDC56 (KU892228); RTDC60 (KU892223); RTDC66 (KU892224)
ST2	4	RTDC44 (KU892245); RTDC45 (KU892246); RTDC 67 (KU892247); RTDC69 (KU892248)
ST3	27	RTDC2 (KU892249); RTDC 3 (KU892250); RTDC6 (KU892251); RTDC10 (KU892252); RTDC 12 (KU892253); RTDC20 (KU892254); RTDC 22 (KU892255); RTDC23 (KU892256); RTDC 24 (KU892257); RTDC25 (KU892258); RTDC 26 (KU892259); RTDC28 (KU892260); RTDC 30 (KU892261); RTDC31 (KU892262); RTDC 33 (KU892263); RTDC34 (KU892264); RTDC 43 (KU892265); RTDC51 (KU892266); RTDC 54 (KU892267); RTDC57 (KU892268); RTDC 58 (KU892269); RTDC59 (KU892270); RTDC 61 (KU892271); RTDC62 (KU892272); RTDC 63 (KU892273); RTDC64 (KU892274); RTDC 65 (KU892275)
ST4	1	RTDC9 (KU892276)
ST1- ST3^a^	9	RTDC13; RTDC15; RTDC18; RTDC21; RTDC47; RTDC48; RTDC53; RTDC68; RTDC70

^a^Subtype-specific sequence-tagged-site (STS) primers

**Fig. 1:**
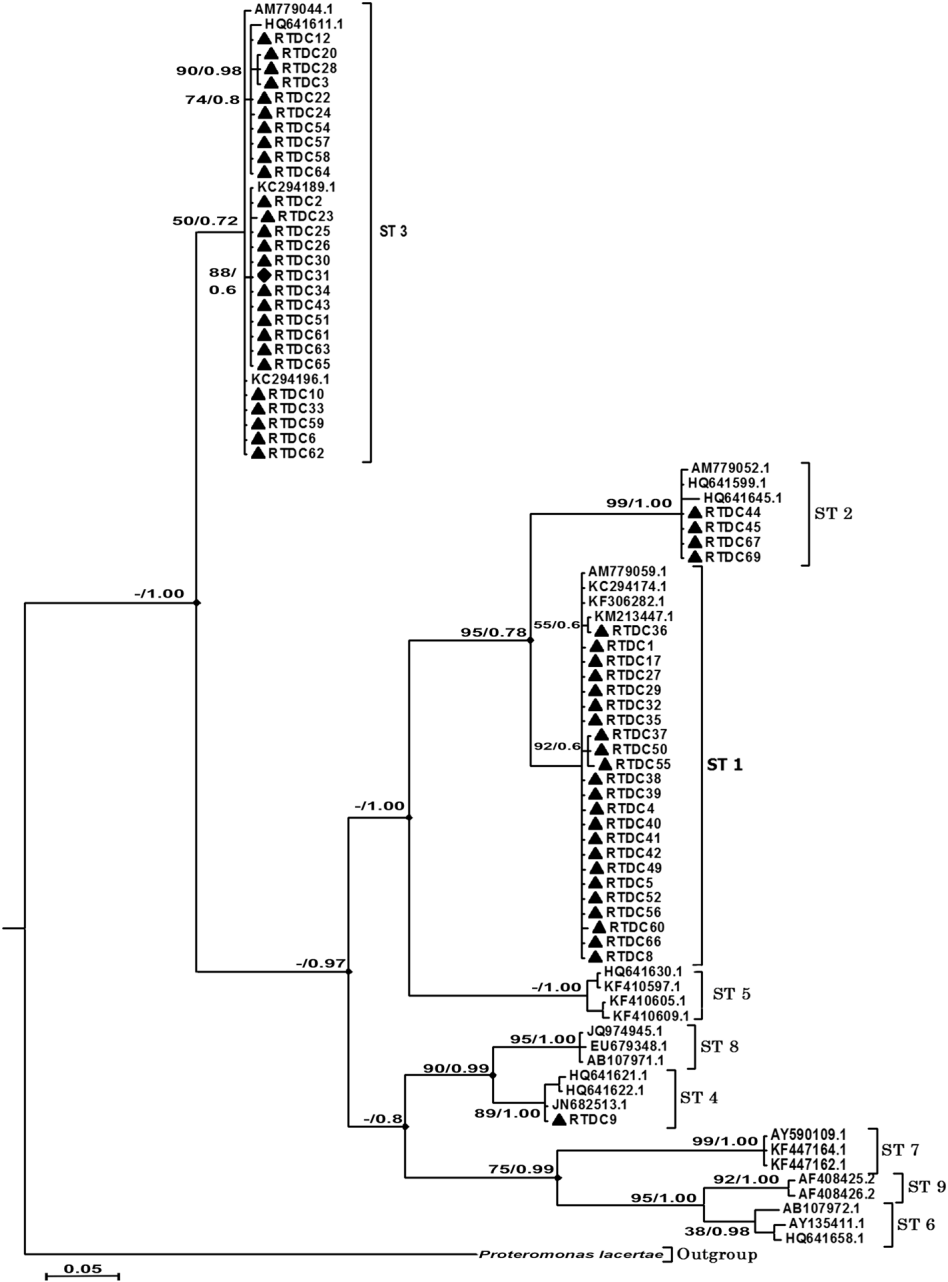
Phylogenetic tree for *Blastocystis* based on SSU rRNA gene sequences. Bayesian Inference (BI) tree reconstructed using the newly generated and GenBank-retrieved partial SSU rRNA gene sequences for *Blastocysts*. The first number associated with each node represents the Maximum Likelihood (ML) bootstrap value followed by the Bayesian Inference posterior probabilities. Black triangles represent Brazilian samples. The scale-bar indicates the expected number of substitutions per site

Phylogenetic analysis of 21 reference sequences using NJ, ML and BI analyses clustered *Blastocystis* subtypes into four monophyletic groups (Fig. 1). Twenty-three isolates clustered with the ST1 reference sequence, four formed a distinct clade with ST2, 27 clustered with ST3, and one clustered with ST4. Intra-strain variation was observed among distinct subtypes: ST1 and ST3 isolates formed small clades within each subtype, indicating extensive genetic variability in the two strains. The posterior probability support in BI analyses had overall higher values than the bootstrap support in ML across the whole tree. Phylogenetic analyses using ML and BI (Fig. 1) and NJ (not shown) indicated that ST1 and ST2 share a common ancestor. The trees also showed that ST6, ST9 and ST7 are related, and that ST8 and ST4 are closely related.

## Discussion

*Blastocystis* is one of the most prevalent unicellular protists found in human fecal specimens [10]. In our study, ST3 was the most prevalent subtype, followed by ST1, ST2, and ST4. In addition, mixed infections were detected in 14% of the samples, similar to the prevalence reported in other countries, which ranged between 2.6–14.3% [6, 12, 19, 46, 49, 62–64]. According to Alfellani et al. [9], single infections are typically reported, whereas mixed infections have been reported in less than half of the studies, at an overall prevalence of only 6%, suggesting a possible under estimation compromising the validity of results and their epidemiological interpretation. Our phylogenetic trees revealed four well-defined clades that grouped isolates into four different subtypes, bearing similarity to those reported in previous studies. In Brazil, even though only three studies have published ST data, the geographical distribution of *Blastocystis* subtypes varies across regions. ST1 was the most prevalent subtype (followed by ST2 and ST3) among an indigenous population from Mato Grosso, Brazil, whereas a study conducted in two small fishing villages situated along the Tietê River in the state of São Paulo, Brazil found a higher incidence of ST3 isolates, followed by ST1 [14, 48, 49].

Globally, subtypes ST1-ST4 have been identified as the most common subtypes in humans [6, 9, 19, 36, 61, 65–70]. Even though it has been suggested that ST3 is the only subtype of human origin [11, 60, 70], it has been detected in several other hosts including pigs, cattle, and non-human primates [3, 9, 65, 71, 72]. Several studies across the world have identified ST3 as the predominant *Blastocystis* subtype [9, 12, 18, 19, 67, 69, 72–80]. Our study identified ST4 in one only sample. This contrasts with previous studies showing that ST4 is common in European and African populations [9, 38, 64, 81–85]. Rodents have been proposed as the reservoir host of this subtype [58, 86–88]. In Colombia, ST4 has been identified in a few humans and non-human primates [85, 86]. However, the molecular epidemiology of *Blastocystis* is incompletely known in other South American countries. To better understand the genetic diversity and determine the prevalence of *Blastocystis* subtypes in South America, further research with larger sample sizes is required.

After ST3, ST1 was the most frequent subtype detected in our study. ST1 has low specificity and is found in a wide range of animals, including dogs, chickens, cattle, pigs and non-human primates [3, 63]. Some studies have suggested that ST1 is linked to zoonotic transmission [89, 90]. *Blastocystis* is transmitted by the fecal-oral route [1] and several studies have suggested that contaminated water may be a source of *Blastocystis* infections [19, 20, 88, 91, 92]. A study conducted in China reported a high infection rate (32.6%) and found that inadequate water intake was a predictor of Blastocystis infection [19]. Also, *Blastocystis* has been detected in samples of soil and drinking water [93]. Overall, many human settlements are characterized by poor hygiene habits, extensive interpersonal contact and increased dependency on care, which provide favorable conditions for the circulation and transmission of parasites and increased susceptibility to parasitic infections. This was revealed by reports of enteric parasitic infections in mental institutions, which indicate that rates of intestinal parasitic infections are higher among institutionalized persons than in the general population, even in developed countries [94]. It should be noted that we analyzed unpreserved stool samples from non-selected adults and identified *Blastocystis* in 46% of family members, compared to a lower figure of 32.5% among institutionalized patients. Therefore, at least in our study population, transmission through cyst-contaminated water appears to be more common than transmission at the care center [87, 88, 90]. The presence of intestinal parasites in the population highlights the need for a more comprehensive epidemiological study in the region to support public policies aimed at educating the public about intestinal parasite prevention. Over the last few years, scientific interest has increased tremendously with respect to the clinical relevance of *Blastocystis* infection and molecular epidemiology.

We should note that the source of DNA (feces *vs* culture) used in PCR may in principle imply a bias in subtyping. We used DNA from cultured *Blastocsytis*, and it is possible that parasite culture could have led to a selection of subtypes of *Blastocystis*. However, this was not observed in a previous study by Stensvold et al. [38] which involved mixed infections. The authors stated that xenic in vitro culture (XIVC) has very little or no impact on subtype distribution or variation within a given specimen. Moreover, the authors recommended that short-term XIVC should be used for cost-effective screening of *Blastocystis* infection from fresh fecal specimens, to generate valid prevalence estimates and to identify isolates. When PCR is based on DNA extracted from feces, the consequence may be false-negative results or decreased PCR efficiency due to a series of factors, including low amount of template DNA in the test sample, inadequate removal of polymerase inhibitors and poor DNA recovery after extraction and purification steps. As a reasonable solution to the presence of inhibitors or degraded target DNA in samples, constitutive genes may be used in parallel assays to evaluate the integrity of the DNA template. PCR is such a powerful tool that even a single molecule of template can be amplified. However, it is difficult to amplify rare templates when they are mixed with similar, but more abundant, templates sharing amplification primers. Nonetheless, no study has evaluated the sensitivity range of PCR using a panel of mixed *Blastocystis* infection samples (multi-ST infections). Moreover, little is known about ST gene targets and how conserved they are both within, and between, subtypes. Had DNA from feces been available for each of the *Blastocystis* samples in our study, it would have been possible to answer some of these important questions.

It has been suggested that pathogenicity may be related to specific *Blastocystis* subtypes, but this association is still inconclusive and often contradictory. Many previous reports on the potential pathogenicity of *Blastocystis* have been limited by a lack of definition of symptoms and more comprehensive investigations able to exclude other infectious agents of diarrhea [91, 95, 96]. Unfortunately, we could not estimate correlations between subtype distributions and symptoms due to the high proportion of asymptomatic individuals, in addition to the fact that individuals had symptoms that could be attributed to other causes (co-infections with pathogenic and commensal intestinal protozoans as well as helminths). In symptomatic individuals not infected by any other protozoans or helminths, the symptoms of *Blastocystis* infection were intermittent episodes of diarrhea, flatulence (in the case of ST2 infection), and flatulence, bloating, and abdominal discomfort but no diarrhea (in the case of ST1, ST2 and ST3). However, other possible causes of clinical symptoms must be considered, such as viral and bacterial infections not evaluated in this study.

## Conclusions

To our knowledge, we conducted the first molecular study of human *Blastocystis* infection in Rio de Janeiro, Brazil. *Blastocystis* isolates were highly genetically divergent. Of the subtypes identified, ST3 was the most common, followed by ST1, ST2 and ST4 (ST4 was identified for the first time in Brazil). However, these results cannot be generalized to the entire Rio de Janeiro population. Further studies are required to determine the distribution of STs in the general population. Nonetheless, our study is a contribution to the understanding of the molecular epidemiology, transmission patterns and genetic diversity of *Blastocystis* at a regional and global scale.

## Abbreviations

AIC: Akaike Information Criterion
BI: Bayesian Inference
BIC: Bayesian Information Criterion
ML: Maximum Likelihood
MLST: multilocus sequence typing
NJ: Neighbor-joining
SSU rDNA: small subunit of the ribosomal RNA gene
ST: subtype
